# Testicular Metabolic Reprogramming in Neonatal Streptozotocin-Induced Type 2 Diabetic Rats Impairs Glycolytic Flux and Promotes Glycogen Synthesis

**DOI:** 10.1155/2015/973142

**Published:** 2015-05-12

**Authors:** L. Rato, M. G. Alves, T. R. Dias, J. E. Cavaco, Pedro F. Oliveira

**Affiliations:** ^1^Health Sciences Research Centre (CICS), Faculty of Health Sciences, University of Beira Interior (UBI), Covilhã, Portugal; ^2^Department of Life Sciences, Faculty of Sciences and Technology and Center for Neurosciences and Cell Biology (CNC), University of Coimbra, Portugal; ^3^Department of Microscopy, Laboratory of Cell Biology, Abel Salazar Institute of Biomedical Sciences (ICBAS), University of Porto, Portugal; ^4^Unit for Multidisciplinary Research in Biomedicine (UMIB), Abel Salazar Institute of Biomedical Sciences (ICBAS), University of Porto (UP), Portugal

## Abstract

Defects in testicular metabolism are directly implicated with male infertility, but most of the mechanisms associated with type 2 diabetes- (T2DM) induced male infertility remain unknown. We aimed to evaluate the effects of T2DM on testicular glucose metabolism by using a neonatal-streptozotocin- (n-STZ) T2DM animal model. Plasma and testicular hormonal levels were evaluated using specific kits. mRNA and protein expression levels were assessed by real-time PCR and Western Blot, respectively. Testicular metabolic profile was assessed by ^1^H-NMR spectroscopy. T2DM rats showed increased glycemic levels, impaired glucose tolerance and hyperinsulinemia. Both testicular and serum testosterone levels were decreased, whereas those of 17*β*-estradiol were not altered. Testicular glycolytic flux was not favored in testicles of T2DM rats, since, despite the increased expression of both glucose transporters 1 and 3 and the enzyme phosphofructokinase 1, lactate dehydrogenase activity was severely decreased contributing to lower testicular lactate content. However, T2DM enhanced testicular glycogen accumulation, by modulating the availability of the precursors for its synthesis. T2DM also affected the reproductive sperm parameters. Taken together these results indicate that T2DM is able to reprogram testicular metabolism by enhancing alternative metabolic pathways, particularly glycogen synthesis, and such alterations are associated with impaired sperm parameters.

## 1. Introduction

Diabetes mellitus (DM) is characterized by hyperglycaemia, resulting from defects in insulin secretion, insulin action, or both. The most prevalent form of DM is type 2 diabetes mellitus (T2DM), comprising up to 95% of all diagnosed diabetic individuals in developed countries [1]. T2DM induces metabolic alterations, disrupting the endocrine system, with a subsequent dysfunction of the hypothalamus-pituitary testicles (HPT) axis [2], that may end up in impairment of the male reproductive health. Although not all diabetic men are infertile, evidences strongly associate T2DM with high prevalence of male subfertility/infertility [3–6] and decreased birth rates [7, 8].

Recently our team reported that diet-induced prediabetes, which is a prodromal stage of T2DM, compromises sperm quality and promotes marked alterations in overall testicular metabolism and ionic homeostasis [9, 10]. The testicles comprise a heterogeneous cell population consisting of both somatic and germ cells. Testicular cells present unique metabolic characteristics, in part due to the existence of the blood-testis barrier. The Sertoli cells (SCs) are responsible for the production of metabolic precursors essential for germ cells development [11, 12]. These cells show a “Warburg-like metabolism” [13], using the external glucose to produce lactate, which is the preferred substrate of developing germ cells [14–17]. Testicular cells in particular SCs take up extracellular glucose via specific glucose transporters (GLUTs), which is then oxidized to pyruvate and promptly reduced to lactate by lactate dehydrogenase (LDH), with a concomitant oxidation of NADH. Once produced, lactate is exported through the monocarboxylate transporter 4 (MCT4) to the intratubular fluid to become available for the developing germ cells. Thus, SCs and germ cells establish a tight metabolic cooperation that is highly dependent on glucose uptake and lactate production [9, 17]. From the physiological point of view the metabolism of carbohydrates, specially glucose, is vital for male reproductive health, so the maintenance of testicular glucose metabolism homeodynamics is of particular relevance; otherwise spermatogenesis is arrested [18, 19]. Hence, the full enlightenment of testicular glucose metabolism and the molecular mechanisms that control it is of extreme importance, since alterations in these mechanisms may be on the basis of male infertility [9, 20, 21]. A role for alternative substrates during diabetic conditions has been suggested (for review see [18]). The use of these alternative substrates, such as glycogen, may act as a compensatory mechanism for fluctuations in insulin and glucose concentrations. Glycogen is a readily mobilized fuel store, which provides the body with a readily available source of energy if blood glucose availability decreases. The presence of glycogen and glycogen metabolism-related enzymes in testicular tissue has already been reported [22–24]. In fact, glycogen seems to play a pivotal role within the testicles, particularly during testicular development [24], where it acts as modulator of germ cell survival [24]. However, the role of glycogen within testicular milieu has been overlooked, particularly under abnormal physiological conditions such as T2DM. Apart from a study showing that diabetic animals present increased testicular levels of uridine diphosphoglucose (UDP-glucose), a glycosyl donor for the initial step of glycogen synthesis [25], no other studies were performed to evaluate the impact of T2DM on testicular glycogen. Herein we aimed to evaluate the effects of T2DM on testicular metabolism, with particular emphasis on glycolytic and glycogen metabolism. We hypothesized that T2DM may impair male fertility by acting in key glycolytic-associated enzymes and transporters, altering testicular metabolism. We also hypothesized that T2DM induces crucial changes in testicular glycogen metabolism.

## 2. Materials and Methods

All chemicals were purchased from Sigma-Aldrich (St. Louis, MO, USA) unless specifically stated. Moloney Murine Leukemia Virus Reverse Transcriptase and deoxynucleotides triphosphates were purchased to NZYTech (Lisbon, Portugal).

### 2.1. Animals

Twelve-three-month-old male Wistar rats (*Rattus norvegicus*) (Charles River Laboratories, Barcelona, Spain) were used in this study. The animals were housed in our accredited animal colony and maintained with food and water* ad libitum* in a constant room temperature (20 ± 2°C) on a 12-hour cycle of artificial lighting. All experiments were performed according to the* Guide for the Care and Use of Laboratory Animals* published by the US National Institutes of Health (NIH Publication number 85–23, revised 1996) and the European directives for the care and handling of laboratory animals (Directive 86/609/EEC).

### 2.2. Experimental Design

Animals were randomly distributed in a control and neonatal-streptozotocin- (n-STZ-) induced T2DM group. Animals from T2DM group were injected with a low dose of STZ to achieve a T2DM model, according to the method described by Iwase and collaborators [26]. In brief, two-day-old male Wistar rats were intraperitoneally injected with STZ (40 mg/kg) freshly diluted in citrate buffer (0.1 M, sodium citrate, pH 4.5). The control group received the vehicle solution in an equivalent volume. All animals were fed with standard chow diet (4RF21 certificate, Mucedola, Italy) and water. Animals' glycaemias were weekly monitored between the 30th and 90th days of age using a glucometer (One Touch Ultra Lifescan-Johnson, Milpitas, CA, USA). After treatment, animals were killed by decapitation. Blood was collected in heparinized tubes for further analysis and testicles were removed, weighed, and stored at −80°C. The levels of glycated hemoglobin (HbA1c) were also determined using A1cNow^+^ meter (Bayer Diabetes Care, USA).

### 2.3. Glucose and Insulin Tolerance Test

At 3 months of age, animals were submitted to a glucose tolerance test, as described by Rato and collaborators [9]. In brief, 14 h before the test, food was removed and the animals were kept in fast. An intraperitoneal (IP) injection with 6 mL of glucose 30% (w/v) per kg of body weight was given to each animal. Blood samples were obtained from the tail and glucose levels measured every 30 min during 2 h. The area under the curve for the glucose tolerance test (AUC_GTT_) was calculated using the trapezoidal rule, as described previously [20].

The animals were also subjected to an insulin tolerance test as described by Holmes and collaborators [27]. In brief, 4 h before the test, food was removed and animals were kept in fast. An IP injection with 0.75 U insulin per kg of body weight was given to each animal. Blood samples were obtained from the tail and glucose levels measured every 30 min during 2 h. The area under the curve for the insulin tolerance test (AUC_ITT_) was calculated using the trapezoidal rule, as described previously [20].

### 2.4. Testosterone, 17*β*-Estradiol, and Insulin Measurement

Hormonal levels were measured as described by Rato and collaborators [9]. In brief, testosterone (T), 17*β*-estradiol (E_2_), and insulin levels were determined using commercial rat EIA kits according to manufacturer instructions. T and E_2_ EIA Kits were purchased from Cayman Chemical Company (Ann Arbor, MI, USA). Insulin ELISA measurement kit was purchased from Mercodia (Uppsala, Sweden). The EIA kits used had detection limits of approximately of 40 *μ*U/mL (for insulin), 6 pg/mL (for T), and 20 pg/mL (for E_2_).

### 2.5. NMR Spectroscopy

A combined extraction of polar and nonpolar metabolites was performed as described by Rato and collaborators [9]. The aqueous phase containing the water-soluble metabolites was lyophilized. ^1^H-NMR spectra were acquired as previously described by Rato and collaborators [9]. Sodium fumarate was used as an internal reference *δ*_H_ [^2^H_2_O] 6.5 [s, 2xCH] to quantify the following metabolites: lactate *δ*_H_ [^2^H_2_O] 1.33 [d, J 6.9 Hz, ^3^CH_3_], alanine 1.45 [d, J −14.36 Hz, ^3^CH_3_], and UDP-glucose 7.97 [d, J 8.1 Hz, CH]. The relative areas of ^1^H-NMR resonances were quantified using the curve-fitting routine supplied with the NUTSpro NMR spectral analysis program (Acorn, CA, USA).

### 2.6. Testicular Glycogen Content

Testicular glycogen content was determined by using a commercial kit (Abnova KA0861, CA, USA) and following the manufacturer's instructions. Glycogen content was expressed as nanomoles of glycogen per milligram of tissue (wet weight).

### 2.7. Quantitative Real-Time PCR

Quantitative real-time PCR (qPCR) was performed to determine glucose transporter 1 (GLUT1), glucose transporter 2 (GLUT2), glucose transporter 3 (GLUT3), phosphofructokinase 1 (PFK1), lactate dehydrogenase A (LDHA), alanine aminotransferase 2 (ALT2), MCT4, muscle glycogen synthase (GYS1), and liver-type glycogen phosphorylase (PGYL) mRNA expression levels. Specific primers were designed for the amplification of target genes and for *β*2-microglobulin which was used as internal control to normalize gene expression (Table 1). qPCR was carried out in an iQ5 system (Bio-Rad, Hercules, CA, USA). Efficiency of the amplification was determined for all primer sets using serial dilutions of cDNA (1, 1 : 5 and 1 : 25). PCR conditions were previously optimized and specificity of the amplicons was determined by melting curves. qPCR amplifications were performed with 1 *μ*L of synthesized cDNA in a 20 *μ*L reaction containing 10 *μ*L Maxima SYBR Green/Fluorescein qPCR Master Mix (Biorad) and 300 nM of sense and antisense primers for each gene. Amplification conditions comprised 5 min denaturation at 95°C, followed by 40 cycles at 95°C for 10 sec, a specific annealing temperature for each gene (Table 1) for 30 sec, and 72°C for 10 sec. Samples were run in triplicate in each PCR assay. Normalized expression values were calculated following the mathematical model proposed by Pfaffl using the formula 2^−ΔΔCt^ [28].

### 2.8. Western Blot

Western Blot procedure was performed as previously described by Simões and collaborators [29]. The resulting membranes were incubated with rabbit anti-GLUT1 (1 : 500, SC-7903, Santa Cruz Biotechnology, Heidelberg, Germany), rabbit anti-GLUT2 (1 : 1000, SC-9117, Santa Cruz Biotechnology, Heidelberg, Germany), rabbit anti-GLUT3 (1 : 1000, ab41525, Abcam, Cambridge, MA, USA), rabbit anti-PFK1 (1 : 500, SC-67028, Santa Cruz Biotechnology, Heidelberg, Germany), rabbit anti-MCT4 (1 : 1000, SC-50329, Santa Cruz Biotechnology, Heidelberg, Germany), rabbit anti-LDH (1 : 10000, ab52488, Abcam, Cambridge, MA, USA), rabbit anti-ALT (1 : 500, SC-99088, Santa Cruz Biotechnology, Heidelberg, Germany), rabbit anti-GYS1 (1 : 100, SC-81173, Santa Cruz Biotechnology, Heidelberg, Germany), and rabbit anti-PYG (1 : 500, SC-66913, Santa Cruz Biotechnology, Heidelberg, Germany). Mouse anti-tubulin (1 : 5000, T9026, Sigma-Aldrich, Rödermark, Germany) was used as protein loading control for testicular tissue. The immunoreactive proteins were separately detected with goat anti-rabbit IgG-AP (1 : 5000, Sc-2007, Santa Cruz Biotechnology, Heidelberg, Germany) or goat anti-mouse IgG-AP (1 : 5000, Sc-2008, Santa Cruz Biotechnology, Heidelberg, Germany). Membranes were reacted with enhanced chemifluorescence detection system (GE Healthcare, Webling, Germany). The densities from each band were obtained using the Quantity One Software (Bio-Rad, Hemel Hempstead, UK), divided by the respective tubulin band densities and then normalized against the respective control animals.

### 2.9. Enzymatic Assays

LDH activity was determined using a commercial assay kit (Promega, Madison, USA) and following the manufacturer's instructions. PFK activity was determined as previously described by Alves and collaborators [30]. ALT was determined by a colorimetric assay as previously described by Mohun and Cook [31]. The attained activities were expressed as fold variation versus the control group.

### 2.10. Epididymal Sperm Parameters

Evaluation of epididymal sperm parameters was performed as previously described by Dias and collaborators [32]. In brief, cauda epididymis was isolated and placed in prewarmed (37°C) Hank's Balanced Salt Solution (pH 7.4), minced with a scalpel blade and the suspension incubated for 5 minutes (37°C). Sperm motility was evaluated by assessing the percentage of motile sperm in 10 random fields, and the average value was used as the total sperm motility. Sperm viability was assessed examining eosin-nigrosin stained epididymal sperm smears. Epididymal sperm concentration was determined using a Neubauer counting chamber. For the assessment of sperm morphology we used standard methods [33]. Sperm morphology was evaluated using Diff-Quick (Baxter Dale Diagnostics AG, Dubinger, Switzerland) stained smears according to the manufacturer's instructions. To be classified as normal, a sperm cell must have a hook-shaped head and no defects of head, neck, or tail. Otherwise, sperm were considered abnormal.

### 2.11. Statistical Analysis

The statistical significances of differences of all experimental data were assessed by Student's *t*-test (Graph Pad Software 6.0, San Diego, CA, USA). All experimental data are shown as mean ± standard error of the mean; *P* < 0.05 was considered significant.

## 3. Results

### 3.1. Streptozotocin-Treated Rats Developed Type 2 Diabetes Mellitus Exhibiting Mild Hyperglycaemia, Glucose Intolerance, and Insulin Resistance

**Figure 1:**
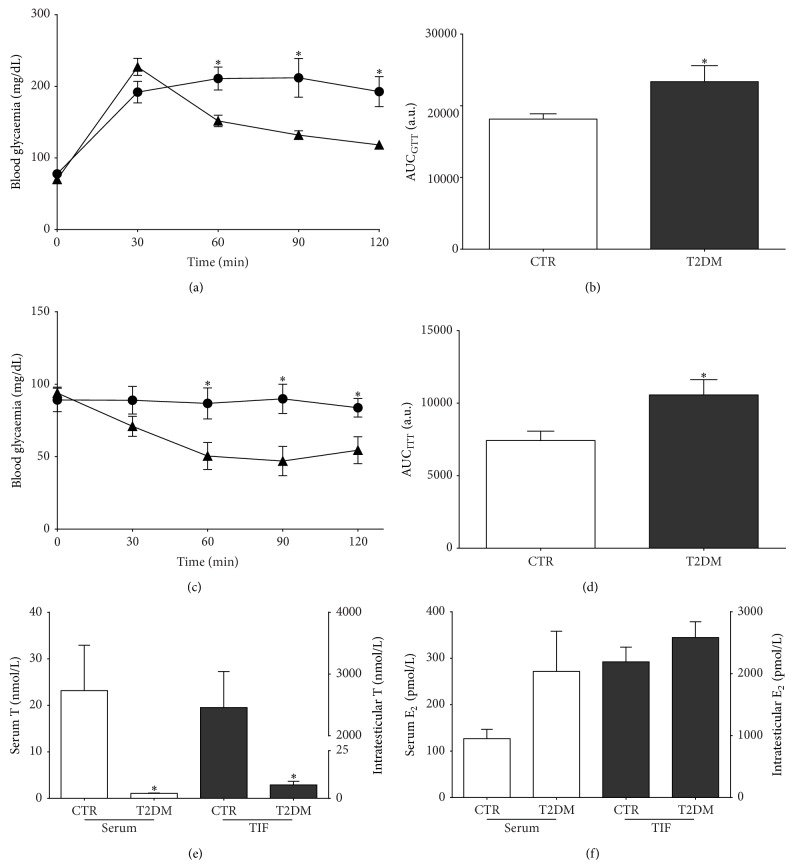
Type 2 diabetes mellitus (T2DM) induces (a and b) glucose intolerance and (c and d) insulin resistance and significantly alters both (e) serum and testicular testosterone levels. (a) Blood glucose levels of the control group (▲) and T2DM group (●) measured during intraperitoneal glucose tolerance test. (b) Area under the curve for the intraperitoneal glucose tolerance test (AUC_GTT_) performed in control and T2DM group rats. (c) Blood glucose levels of the control group (▲) and T2DM group (●) measured during intraperitoneal insulin tolerance test. (d) Area under the curve for the intraperitoneal insulin tolerance test (AUC_ITT_) performed in control and T2DM group rats. (e) Testosterone levels in intratesticular interstitial fluid and serum of control and T2DM group rats. (f) 17*β*-Estradiol levels in intratesticular interstitial fluid and serum of control and T2DM group rats. Results are presented as mean ± SEM of six independent experiments, corresponding to six animals/group. ∗Significantly different relative to control (*P* < 0.05).

After three months of age average glycaemic values were significantly increased (by 26%) in n-STZ-treated animals (126.0 ± 1 mg/dL), when compared to control group (99.0 ± 1 mg/dL; Table 2). Blood HbA1c levels were also significantly increased (by 17%) in n-STZ-treated animals (5.60 ± 0.07%) when compared to control group (4.80 ± 0.02%; Table 2). Together, these results prefigure a prolonged state of hyperglycaemia and subsequent impaired glucose metabolism. Indeed, the results attained for the glucose tolerance test show that blood glycaemia of n-STZ-treated animals increased during the 120 min of the test (Figure 1(a)), indicating the development of glucose intolerance. This can be seen by the significantly increased (by 29%) AUC_GTT_ values in n-STZ-treated animals (23364 ± 2231 arbitrary units (a.u.)) when compared to animals from the control group (18153 ± 735 a.u.) (Figure 1(b)). These results led us to investigate the insulin responsiveness status, so we performed an IP insulin tolerance test. Our results showed that n-STZ-treated animals did not respond to insulin (Figure 1(c)) as observed by the significant increase of AUC_ITT_ (by 30%) in n-STZ-treated animals (10570 ± 1054 a.u.), when compared with rats from the control group (7420 ± 657 a.u.) (Figure 1(d)), illustrating that these rats developed insulin resistance. The higher levels of fasting insulin (increased by 21%) observed in n-STZ-treated animals (Table 2) are consistent with these results, corroborating that these rats developed insulin resistance. Altogether these characteristics clearly illustrate that the n-STZ-treated rats developed type 2 diabetes (T2DM group).

### 3.2. T2DM Decreases Serum and Testicular Testosterone Levels

We evaluated the levels of the sex steroid hormones (T and E_2_) in serum and testicular environment, since they are pivotal for several events that control spermatogenesis, including testicular metabolism [17]. T serum concentration was significantly decreased (by 95%) in T2DM group (1.06 ± 0.09 nmol/L) when compared with the control group (23.16 ± 9.76 nmol/L) (Figure 1(e)). On the other hand, serum E_2_ levels were not altered in the animals that developed T2DM (272 ± 87 pmol/L) when compared to control (127 ± 20 pmol/L) (Figure 1(f)). Testicular cells are bathed by testicular interstitial fluid (TIF) and the establishment of an appropriate fluid is crucial for an adequate hormonal control of spermatogenesis. In this fluid, sex steroid levels are 100- to 1000-fold higher than in serum, as observed in a previous study [9]. T levels present in TIF were significantly decreased by 348% in T2DM group animals (7 ± 2 nmol/L) when compared with animals from the control group (2457 ± 583 nmol/L) (Figure 1(e)). Contrastingly, T2DM animals did not exhibited significantly altered E_2_ concentrations in TIF (2585 ± 255 pmol/L) when compared to control (2192 ± 239 pmol/L) (Figure 1(f)).

### 3.3. T2DM Increases GLUT1/GLUT3 Levels and PFK Activity in Rat Testicles

**Figure 2:**
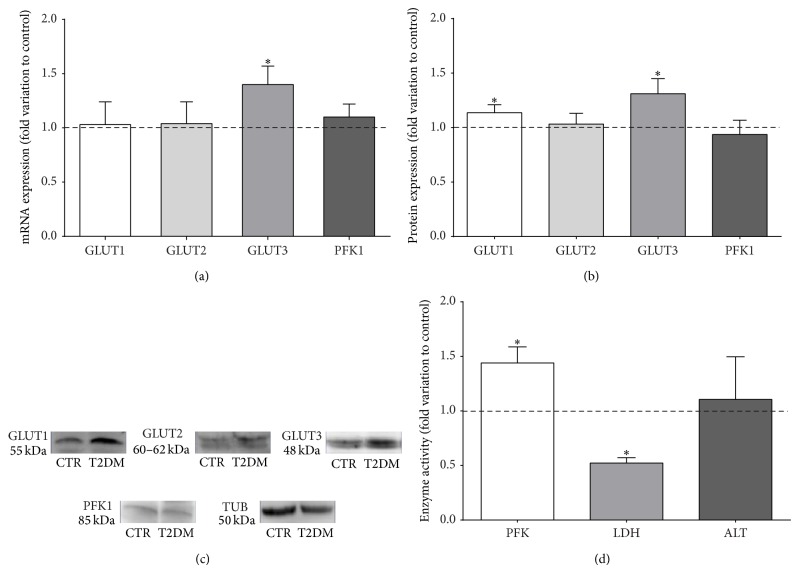
Type 2 diabetes mellitus (T2DM) modulates the (a) mRNA, (b) protein levels, and (d) activity of glycolytic enzymes and transporters. (a) Pooled data of independent experiments, indicating the fold variation of glucose transporters (GLUT1, GLUT2, and GLUT3) and of phosphofructokinase 1 (PFK1) mRNA levels found in testicles of T2DM rats when compared with the control rats (dashed line). (b) Pooled data of independent experiments, indicating the fold variation of GLUT1, GLUT2, GLUT3, and PFK1 protein levels found in testicles of T2DM rats when compared with the control rats (dashed line). (c) Illustrative Western Blot experiment. (d) Pooled data of independent experiments, indicating the fold variation of PFK1, lactate dehydrogenase (LDH), and alanine transaminase (ALT) enzymatic activities in testicles of T2DM rats when compared with the control rats (dashed line). Results are expressed as mean ± SEM (*n* = 6 for each condition). ∗Significantly different relative to control (*P* < 0.05).

Glucose metabolism is pivotal for the normal occurrence of spermatogenesis. Its uptake from the extracellular medium is a rate-limiting step for glucose metabolism. Hence we evaluated the effects of T2DM on the expression of the most relevant glucose membrane transporters in testicles (GLUT1, GLUT2, and GLUT3). No significant differences were observed in testicular GLUT1 transcript levels between animals from both groups (Figure 2(a)), while GLUT1 protein in animals from T2DM group was significantly increased in 13% (1.13 ± 0.07-fold variation), when compared with the control group (Figure 2(b)). Concerning GLUT2 expression, no alteration was observed in both transcript and protein levels of T2DM animals when compared with the control group (Figures 2(a) and 2(b)). GLUT3 mRNA expression was significantly increased by 40% in T2DM group (1.40 ± 0.17-fold variation to control) (Figure 2(a)) and was followed by a 31% increase in protein levels (1.31 ± 0.13-fold variation to control) (Figure 2(b)). After being internalized, glucose is metabolized via glycolysis, in which the irreversible conversion of fructose 6-phosphate to fructose 1,6-bisphosphate by PFK is a key control point. We evaluated the effects of T2DM in PFK1 levels (widely expressed in testicular tissue) and observed no alteration in both transcripts and protein levels of PFK1. Nevertheless, when we assessed the activity of PFK it was significantly increased by 44% in the testicular tissue of T2DM rats (1.44 ± 0.14-fold variation), when compared with testicular tissue of rats from the control group (Figure 2(d)).

### 3.4. T2DM Causes a Reduction of Testicular Lactate Levels by Decreasing LDH Activity

In testicular tissue the majority of glucose is converted into lactate, which is not a “waste” end-product of glycolysis but a critical “fuel” for germ cells development. Thus, we evaluated the effects of T2DM on the testicular glycolytic metabolic profile. Our results show that testicular lactate content was significantly decreased by 50% in rats from T2DM group (1.45 ± 0.30 nmol/mg tissue), when compared to rats from the control group (2.90 ± 0.60 nmol/mg tissue) (Table 3).

**Figure 3:**
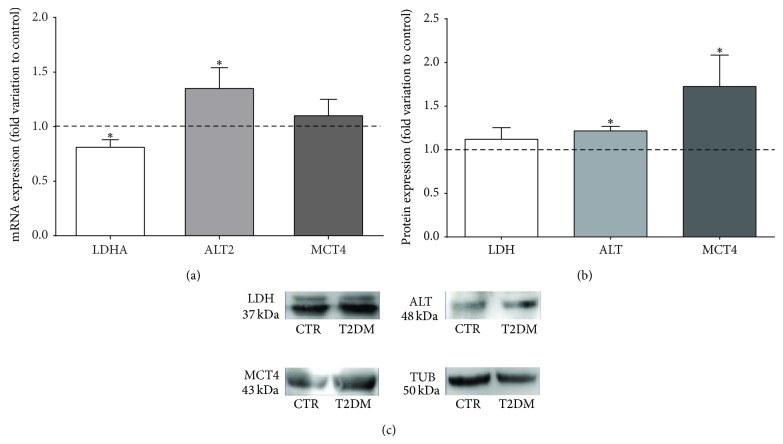
Type 2 diabetes mellitus (T2DM) modulates the expression of lactate production-related enzymes and transporter. (a) Pooled data of independent experiments, indicating the fold variation of lactate dehydrogenase (LDH), alanine transaminase 2 (ALT2), and monocarboxylate transporter 4 (MCT4) mRNA levels found in testicles of T2DM rats when compared with the control rats (dashed line). (b) Pooled data of independent experiments, indicating the fold variation of LDH, ALT, and MCT4 protein levels found in testicles of T2DM rats when compared with the control rats (dashed line). (c) Illustrative Western Blot experiment. Results are expressed as mean ± SEM (*n* = 6 for each condition). ∗Significantly different relative to control (*P* < 0.05).

Since testicular lactate content was significantly reduced, we evaluated the effects of T2DM on the expression and activity of LDH. When we assessed the LDHA transcript levels (which is highly expressed in the lactate-producing SCs), we observed a significant reduction (by 19%) in the testicles of rats from the T2DM group (0.81 ± 0.07-fold variation) when compared with rats from the control group (Figure 3(a)). However, the overall protein levels were not altered in the testicles of rats from both groups (Figure 3(b)). We further evaluated LDH activity on the testicular tissue of animals from both groups and observed a significant decrease (by 48%) in the testicles from T2DM rats (0.52 ± 0.05-fold variation; Figure 2(d)), as compared with rats from the control group.

Once produced, in order for lactate to reach the developing germ cells it must be exported from SCs MCT4. When we evaluated the testicular expression of MCT4, we observed no differences between animals from T2DM and control groups concerning mRNA levels (Figure 3(a)). However, MCT4 protein levels were significantly increased (72%) in the testicular tissue of T2DM rats (1.72 ± 0.35-fold variation) when compared with rats from control group (Figure 3(b)).

### 3.5. T2DM Slightly Increases the Testicular Content of Alanine, Altering the Lactate/Alanine Ratio

Lactate is converted from pyruvate, which is at a crossroad of several metabolic pathways. Pyruvate is an intermediary metabolite that can be reversibly converted either to lactate (by LDH) or to alanine (by alanine aminotransferase (ALT)). Following the observed decrease in testicular lactate content of T2DM rats, we further evaluated testicular alanine levels. We found it to be slightly (but not significantly) increased in T2DM rats (0.73 ± 0.20 nmol/mg tissue; Table 3), as compared with rats from the control group (0.70 ± 0.20 nmol/mg tissue).

We then assessed the mRNA levels of the main testicular ALT isoform (ALT2). Our results show a significant increase (by 35%) in T2DM rats (1.35 ± 0.19-fold variation to control) (Figure 3(a)). Additionally, we observed that the overall testicular protein levels of ALT were also significantly increased by 21% in T2DM rats (1.21 ± 0.05-fold variation to control) (Figure 3(b)). However, when we evaluated the activity levels of ALT in testis we found no significant differences between both groups (Figure 2(d)). Importantly, the lower levels of lactate together with the slight increase in the alanine levels detected in the testicular tissue of T2DM rats illustrate a decrease of the lactate/alanine ratio (Table 3) (2.70 ± 0.40), as compared with the control group (3.70 ± 0.30).

### 3.6. T2DM Enhances Testicular Glycogen Deposition, Modulating the Expression of Glycogen-Associated Enzymes

**Figure 4:**
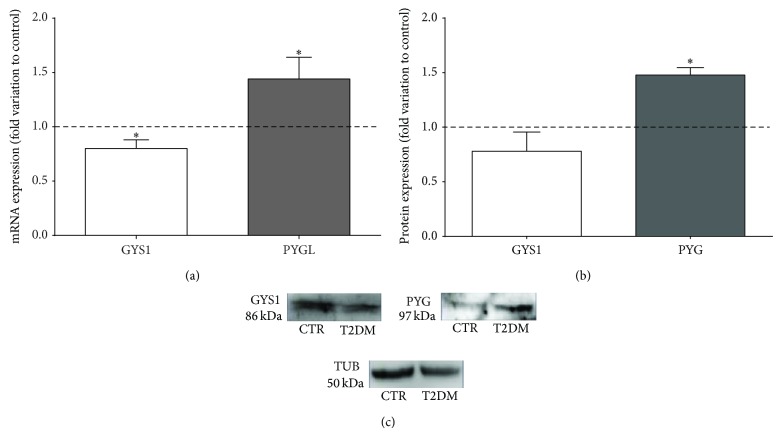
Type 2 diabetes mellitus (T2DM) modulates the expression of glycogen metabolism-related enzymes. (a) Pooled data of independent experiments, indicating the fold variation of glycogen synthase 1 (GYS1) and glycogen phosphorylase L (PYGL) mRNA levels found in testicles of T2DM rats when compared with the control rats (dashed line). (b) Pooled data of independent experiments, indicating the fold variation of GYS1 and PYG protein levels found in testicles of T2DM rats when compared with the control rats (dashed line). (c) Illustrative Western Blot experiment. Results are expressed as mean ± SEM (*n* = 6 for each condition). ∗Significantly different relative to control (*P* < 0.05).

We evaluated the testicular content of glycogen and of its precursor monomers UDP-glucose. We observed that glycogen levels in the testicles of T2DM rats were significantly increased by 26% (0.41 ± 0.10 nmol/mg tissue), as compared with the control group (0.25 ± 0.03 nmol/mg tissue; Table 3). We also observed that UDP-glucose testicular content in T2DM rats was significantly increased by 12.5% when compared with the control group, from 0.48 ± 0.01 nmol/mg tissue to 0.54 ± 0.01 nmol/mg tissue (Table 3). We further evaluated the effects of T2DM on testicular glycogen metabolism by assessing the expression levels of key rate-limiting enzymes involved in testicular glycogen metabolism. The mRNA levels of GYS1 (the main GYS isoform expressed in the testicles) were significantly decreased by 20% in the testicles of T2DM rats (0.80 ± 0.01-fold variation) when compared to rats of the control group, while its protein levels were not significantly altered (Figures 4(a) and 4(b)).

On the other hand, when we evaluated the testicular expression levels of glycogen phosphorylase (PYG), a key enzyme responsible for glycogen degradation, we found that these were significantly altered by T2DM. We assessed PYGL transcript levels (the isoform preferentially expressed in the testicles) and overall PYG protein levels. Our results show that PYGL mRNA levels were significantly increased by 44% in T2DM rats (1.44 ± 0.20-fold variation) when compared with rats from the control group (Figure 4(a)). The overall PYG protein levels were also significantly increased by 48% in the testicles of T2DM rats (1.48 ± 0.06-fold variation to control) (Figure 4(b)).

### 3.7. T2DM Affects Epididymal Sperm Quality

T2DM is known to affect sperm quality parameters, such as concentration, motility, viability, and morphology [5]. As could be expected, our results showed that T2DM rats presented alterations in specific epididymal sperm parameters when compared with rats from the control group. Although the sperm concentration in control rats was 4.10 ± 0.40 × 10^7^ cell/mL, which was not significantly different from T2DM rats (4.70 ± 0.40 × 10^7^ cell/mL), spermatozoa total motility of T2DM rats was significantly lower (73.2 ± 1.0%) relatively to control rats (78.2 ± 0.9%). Furthermore, when we evaluated epididymal spermatozoa viability, control rats showed a significantly higher viability (74.0 ± 0.2%) than T2DM rats (51.0 ± 1.0%; Table 4). When assessing sperm morphology, our results showed that the percentage of abnormal spermatozoa in control group was 38.0 ± 3.0% and T2DM rats presented a significantly higher percentage of abnormal spermatozoa (49.0 ± 3.0%; Table 4).

## 4. Discussion

T2DM is the most prevalent form of DM, characterized by insulin resistance and impairment of whole body metabolism [1]. Several animal models have been developed to study the mechanisms involved in the pathophysiology of the various comorbidities associated with T2DM [34]. In this study, we used a n-STZ-induced T2DM animal model that displays the typical characteristics of the early stages of T2DM. STZ is a widely used diabetogenic agent, which produces a selective toxic effect on pancreatic *β*-cells [35]. When administrated to rats at low doses during postnatal age they are reported to develop T2DM [26]. In our study the n-STZ-treated animals showed mild hyperglycaemia and impaired glucose tolerance, as observed by the significant increase in the AUC_GTT_ values. In fact, when subjected to a glucose tolerance test, their glycaemia levels remained within the interval 140–200 mg/dL 2 hours after glucose load. This is consistent with the observed increase of HbA1c values in T2DM rats, which were in accordance with others [26, 36]. HbA1c is a marker of cumulative glycaemic exposure over a preceding of 8 to 12 weeks and a strong predictor of the development of DM [1]. Insulin intolerance (resistance) is also closely associated with T2DM [1] and was also observed in our animal model. Furthermore, plasma insulin levels were also increased. This may be explained as a compensatory mechanism in order to maintain normoglycemia to face the detected insulin intolerance in T2DM rats [37]. Hyperinsulinemia presented by T2DM rats is also concomitant with the marked insulin intolerance observed in these animals. Altogether, the displayed characteristics of STZ-treated animals clearly indicate that a T2DM condition was attained in these rats.

T2DM rats presented insulin intolerance that has been associated with disruption of the HPT axis, leading to an imbalance in the levels of sex steroid hormones [38]. Insulin is essential for the normal function of the reproductive axis. Deficiency in this hormone has been directly associated with a decrease in T secretion by Leydig cells [39, 40]. In this context, we evaluated sex steroids levels and observed a significant decrease in both testicular and serum T levels in T2DM rats. Undoubtedly, insulin intolerance compromises testicular T production and consequently whole body T levels, as previously observed in insulin resistant individuals [39]. Concerning the E_2_ levels, we did not observe significant differences at both testicular and serum levels between groups, which is in accordance with other reports [41]. Unaltered E_2_ levels may be explained not only by the unchanged levels and/or enzyme activity of aromatase observed in early stages of DM [41], but also by the contribution of peripheral tissues, such as adipose tissue, bone, and skin [42]. Therefore the T2DM promoted by STZ might not be sufficiently advanced to induce significant differences in E_2_ levels that are known to occur in more severe stages of the disease [38, 43].

The endocrine disruption observed in T2DM rats is reflected in whole body metabolism and may also disturb testicular energy metabolism and male reproductive function [17, 44, 45]. Testicular tissue consists of a heterogeneous population of somatic and germ cells, where germ cells are dependent on the nutritional support provided by SCs and DM alters this metabolic cooperation. It has been discussed that the altered metabolism within testicular environment is closely associated with decreased male fertility (for review see [46]). Previous studies from our team showed that, in a prodromal stage of T2DM, known as prediabetes, testicular cells are able to adapt their metabolism to promote an adequate environment for germ cell development [9]. Glucose uptake is enhanced in testicular tissue of prediabetic animals, as well as PFK activity, favoring a high glycolytic flux [9]. Moreover, the testicular expression levels of proteins involved in lactate production and transport were also found to be enhanced in prediabetic animals, resulting in higher amounts of testicular lactate [9]. Like prediabetic rats, our T2DM rats presented increased testicular expression levels of GLUT1 and GLUT3, favoring the glucose uptake. This was accompanied by increased activity of PFK1, thus showing that the two rate-limiting steps of glycolysis are not affected by T2DM which illustrates that the high testicular glycolytic flux observed in the testicles of prediabetic animals may also occur in the testicles of T2DM. In sum, testicles metabolize glucose at high rates not only in prodromal stages but also when T2DM is already established. However, we observed that testicular lactate production is reduced by T2DM. In the present study, the main contributor to the lower lactate content detected in testicles of T2DM rats seems to be the decrease of LDH activity. Moreover, lactate export is not impaired by T2DM, since MCT4 levels were increased in T2DM rats. Concurrent results have been described in a previous study using a spontaneous T2DM animal model [47]. Those authors reported that 3-month-old Goto-Kakizaki rats, which constitute an important model to study the initial events of DM development (since at early age these animals do not exhibit the severe complications associated with the disease) [48], showed reduced testicular lactate production, illustrating the existence of testicular metabolic adaptations according to the degree of severity of T2DM.

The present data illustrates that testicular glycolytic flux, which ends up in lactate production, is compromised in T2DM rats. Glucose taken up by testicular cells is not converted into lactate as efficiently, being most probably redirected to other metabolic pathways. Pyruvate, the end-product of glycolysis, can be used as a precursor metabolite for several metabolic pathways. Nevertheless, within the testicular milieu it is preferentially converted to lactate by LDH or alanine by ALT [17]. We found that alanine levels were slightly (but not significantly) increased in T2DM rats, which was associated with a slight (nonsignificant) increase of ALT activity. This slight increase in alanine levels together with the lower levels of lactate led to a decrease in testicular lactate/alanine ratio in T2DM rats. This lactate/alanine ratio is often used as an index of the redox state of tissues [49], because it reflects the NAD^+^/NADH ratio, since the conversion of pyruvate to alanine is coupled with reoxidation of NADH into NAD^+^. NAD^+^/NADH is directly implicated with energy metabolism constituting a metabolic node well suited for integration of energy metabolism. The decreased testicular lactate/alanine ratio illustrates that, in the present conditions, the feed-forward pathway in which LDH fuels glyceraldehyde 3-phosphate dehydrogenase with NAD^+^ is not favored, which may eventually end up in compromised glycolytic pathway.

Kim and collaborators [50] have described an association between alterations in glucose metabolism and decreased sperm quality in diabetic mice models. Although those authors used a distinct type 1 diabetes rodent model, they observed a decrease in sperm parameters that they correlated with an abnormal metabolic activity, which could also be exacerbated by the local autoimmune mechanism known to occur in this pathological state [5, 51]. In that study, Kim and collaborators [50] reported an alteration of expression of specific GLUTs involved either in the supply of substrates to the pentose phosphate pathway known to occur in the sperm head (for review see [52]) or in the uptake of fructose from the seminal plasma (see review [53]), pointing to possible impairment in the maturation and fertilization events and not in spermatogenesis [50]. No information was given concerning the other rate-limiting steps of the glycolytic flux and production of lactate.

Still, our results demonstrate that T2DM condition altered testicular GLUT's expression, favoring the glucose uptake, while the production of lactate, the key substrate for developing germ cells, was compromised. These evidences suggested that, in our animal model, T2DM induced a testicular metabolic reprogramming, promoting distinct glucose metabolic pathways and/or substrate preferences. Bearing in mind that glycogen metabolism plays a preponderant role in T2DM, contributing to high glucose disposal (for review see [9, 54]) and that testicles readily synthetize and store glycogen [24] we hypothesized that, under T2DM conditions, testicular glycogen may represent an alternative metabolic pathway and contribute to adaptive metabolic mechanisms. We evaluated testicular glycogen content and observed a significant increase in T2DM rats. T2DM animals present several conditions that favor glycogenesis such as (1) elevated levels of insulin, which stimulate glycogen synthesis through inactivation of glycogen synthase kinase 3 (GSK-3) that is known to maintain GYS1 in an activated form; (2) hyperglycaemia, which increases the blood-to-testicles glucose availability, exerting an allosteric activation on GYS1 via glucose-6-phosphate (accelerating glycogen synthesis) [55, 56]; (3) increased testicular levels of UDP-glucose, an active intermediate in the synthesis of glycogen [25]. As glycogen synthase is the rate-limiting enzyme in glycogen synthesis, we evaluated testicular GYS1 levels. We found no significant alterations in GYS1 protein levels of T2DM rats, although a significant decrease was observed in GYS1 mRNA levels. These divergent results concerning mRNA and protein levels may be due to factors associated with regulatory mechanisms in gene expression, such as mRNA retention in the nucleus, processing, stability, and half-life time of mRNA molecule [57]. The simultaneous increase in testicular glycogen amount and overall PYG levels seems to be somewhat contradictory. A possible explanation may rely on the increased levels of sugar nucleotides, such as UDP-glucose, which is the immediate precursor in glycogen synthase reaction. Increased levels of UDP-glucose were detected in the testicles of T2DM rats illustrating the ability of testicular tissue to synthesize glycogen. This increase of UDP-glucose and glycogen content in tissues promoted by T2D was also reported in the heart [58, 59]. The regulation of glycogen metabolism is complex and several enzymes taking part in this process allosterically respond to metabolites and/or hormones. It has been proposed that the increased cardiac glycogen synthesis observed in STZ-induced diabetic rats [60] is due to a metabolic reprogramming of cardiac tissue, in which the increased glucose influx together with the reduced glucose oxidation augments the cardiac content of UDP-glucose [61]. This effect with the combined activation of glycogen synthase increases cardiac glycogen storage. Our results support that this phenomenon may also occur in testicles. The increased GLUT1 and GLUT3 expression, in opposition to the low lactate production and together with the higher levels of UDP-glucose in T2DM rats, illustrate that T2DM stimulates glycogen accumulation.

Despite the ability of testicles to adopt alternative pathways, the altered glucose utilization and the glycogen accumulation observed in testicles of T2DM rats were concurrent with the impairment of sperm parameters. In fact, although no changes were observed in sperm concentration of T2DM rats, the percentage of abnormal sperm was significantly increased. A side effect of hyperglycaemia and altered testicular bioenergetics is the increase of oxidative stress and reactive oxygen species overproduction within testicular environment [20], which contributes to abnormal sperm morphology. Thus, our results are consistent with previous studies [47, 62], illustrating that testicular metabolic alterations induced by T2DM are closely associated with a decrease in male fertility. However, one cannot discard that subnormal androgenic status prevailing in T2DM animals also contributes to the impairment of secretory activity of epididymal epithelium which may contribute to the observed decline of sperm parameters [63].

In conclusion, our results clearly suggest that T2DM significantly alters testicular glycolytic profile and glycogen metabolism and such alterations are associated with each other and lead to the impairment of sperm quality; nevertheless the underlying molecular mechanisms that lead to decreased sperm parameters must be fully elucidated. Further knowledge on the functioning and regulation of these mechanisms will be essential to provide new insights into effects of T2DM on male fertility.

## Figures and Tables

**Table 1:** Oligonucleotides and cycling conditions for PCR amplification of GLUT1, GLUT2, GLUT3, PFK1, LDHA, MCT4, ALT2, GYS1, PYGL, and *β*2MG.

Gene	Sequence (5′-3′)	AT (C°)	Accession number
GLUT1	Sense: TCCGGCGGGAGACGCATAGT	61	NM_138827.1
	Antisense: CCCGCATCATCTGCCGACCC		

GLUT2	Sense: GGGTTCCTTCCAGTTCGGAT	60	NM_012879.2
	Antisense: TCGTATGTGCTGGTGTGACT		

GLUT3	Sense: GCGCAGCCCTTCCGTTTTGC	63	NM_017102.2
	Antisense: CCCCTCGAAGGCCCGGGTAA		

PFK1	Sense: GAGTGCTGACAAGCGGCGGT	61	NM_013190.4
	Antisense: GTGGCCCAGCACGGTCACTC		

LDHA	Sense: GCGCAGCCCTTCCGTTTTGC	63	NM_017025.1
	Antisense: CCCCTCGAAGGCCCGGGTAA		

ALT2	Sense: TGAGGTAATCCGAGCCAACA	60	NM_001012057.1
	Antisense: CACGTCCTCTCGGATACAGT		

MCT4	Sense: ATGTGGGCATGGCGTGTGCC	66	NM_001013913.1
	Antisense: CCCAGCCATGGCAGCTCGAA		

GYS1	Sense: CAGCTATGGGACACAGCCAA	60	NM_001109615.1
	Antisense: TTCGTCGGATGGTGGTCAAG		

PYGL	Sense: CTCCCAATCAGCCAGACCTC	60	NM_022268.1
	Antisense: GGAAGGCTCCATGTTCCAGA		

*β*2MG	Sense: ATGAGTATGCCTGCCGTGTG	60	NM_012512.2
	Antisense: CAAACCTCCATGATGCTGCTTAC		

Legend: AT: annealing temperature; ALT2: alanine aminotransferase 2; GLUT1: glucose transporter 1; GLUT2: glucose transporter 2; GLUT3: glucose transporter 3; GYS1: muscle glycogen synthase; LDHA: lactate dehydrogenase A; MCT4: monocarboxylate transporter 4; PFK1: phosphofructokinase 1; PYGL: liver-type glycogen phosphorylase; *β*2MG: beta 2 microglobulin.

**Table 2:** Average values of the animals weight, glycaemia, HbA1c, and insulin levels measured in animals from the control (CTR) and T2DM group after three months of age.

Parameters	CTR	T2DM
Body weight (g)	347 ± 8	363 ± 11
Average Glycaemia (mg/dL)	99 ± 1	126 ± 1^∗^
HbA1c (%)	4.80 ± 0.02	5.60 ± 0.07^∗^
Insulin (*μ*U/mL)	4.3 ± 0.3	5.2 ± 0.4^∗^

Legend: CTR: control; HbA1c: glycated hemoglobin; T2DM: type 2 diabetes mellitus. Results are expressed as mean ± SEM (*n* = 6 for each condition). ^∗^Significantly different relative to control (*P* < 0.05).

**Table 3:** Metabolite content of testicular tissue in animals from the control (CTR) and T2DM group.

Metabolites (nmol/mg tissue)	CTR	T2DM
Lactate	2.90 ± 0.60	1.45 ± 0.30^∗^
Alanine	0.70 ± 0.20	0.73 ± 0.20
Glycogen	0.25 ± 0.03	0.41 ± 0.10^∗^
UDP-Glucose	0.48 ± 0.01	0.54 ± 0.01^∗^

Lactate/Alanine ratio	3.70 ± 0.30	2.70 ± 0.40^∗^

Legend: CTR: control; T2DM: type 2 diabetes mellitus. Results are expressed as mean ± SEM (*n* = 6 for each condition). ^∗^Significantly different relative to control (*P* < 0.05).

**Table 4:** Epididymal sperm concentration, motility, viability, and morphology in animals from the control (CTR) and T2DM group.

Sperm parameters	CTR	T2DM
Concentration (×10^7^ cell·mL^−1^)	4.10 ± 0.40	4.70 ± 0.40
Motility (%)	78.20 ± 0.90	73.20 ± 1.00^∗^
Viability (%)	74.00 ± 2.00	51.00 ± 1.00^∗^
Morphology (% abnormal spermatozoa)	38.00 ± 3.00	49.00 ± 3.00^∗^

Legend: CTR: control; T2DM: type 2 diabetes mellitus. Results are expressed as mean ± SEM (*n* = 6 for each condition). ^∗^Significantly different relative to control (*P* < 0.05).

## Abbreviations

^1^H-NMR: Proton nuclear magnetic resonance
a.u.: Arbitrary units
ALT: Alanine aminotransferase
ALT2: Alanine aminotransferase 2
AUC_GTT_: Area under the curve for the glucose tolerance test
AUC_ITT_: Area under the curve for the insulin tolerance test
DM: Diabetes mellitus
E_2_: 17*β*-Estradiol
G-6-P: Glucose-6-phosphate
GLUT1: Glucose transporter 1
GLUT2: Glucose transporter 2
GLUT3: Glucose transporter 3
GLUT8: Glucose transporter 8
GLUTs: Glucose transporters
GSK-3: Glycogen synthase kinase 3
GYS1: Muscle glycogen synthase
HbA1c: Glycated hemoglobin
HPT: Hypothalamus-pituitary testicles
IP: Intraperitoneal
LDH: Lactate dehydrogenase
LDHA: Lactate dehydrogenase A
MCT4: Monocarboxylate transporter 4
PFK1: Phosphofructokinase 1
PYG: Glycogen phosphorylase
PYGB: Brain-type glycogen phosphorylase
PYGL: Liver-type glycogen phosphorylase
qPCR: Quantitative real-time PCR
SCs: Sertoli cells
n-STZ: Neonatal streptozotocin
T: Testosterone
T2DM: Type 2 diabetes mellitus
TIF: Testicular interstitial fluid
UDP-glucose: Uridine diphosphoglucose.
